# An engineered, quantifiable *in vitro* model for analysing the effect of proteostasis-targeting drugs on tissue physical properties

**DOI:** 10.1016/j.biomaterials.2018.08.041

**Published:** 2018-11

**Authors:** Sandra Loaiza, Silvia A. Ferreira, Tamara M. Chinn, Alex Kirby, Elena Tsolaki, Camilla Dondi, Katarzyna Parzych, Adam P. Strange, Laurent Bozec, Sergio Bertazzo, Martin A.B. Hedegaard, Eileen Gentleman, Holger W. Auner

**Affiliations:** a Cancer Cell Protein Metabolism Group, Department of Medicine, Imperial College London, London W12 0NN, UK; b Centre for Craniofacial and Regenerative Biology, King's College London, London SE1 9RT, UK; c Department of Medical Physics and Biomedical Engineering, University College London, London WC1E 6BT, UK; d Biomaterials and Tissue Engineering, Eastman Dental Institute, University College London, London WC1X 8LD, UK; e Department of Chemical Engineering, Biotechnology and Environmental Technology, University of Southern Denmark, 5230 Odense M, Denmark; f Faculty of Dentistry, University of Toronto, 124 Edward Street, Toronto, ON M5G 1G6, Canada

**Keywords:** Proteostasis, VCP/p97, Raman spectroscopy, Cancer diagnosis and therapy, Atomic force microscopy, Proteasome

## Abstract

Cellular function depends on the maintenance of protein homeostasis (proteostasis) by regulated protein degradation. Chronic dysregulation of proteostasis is associated with neurodegenerative and age-related diseases, and drugs targeting components of the protein degradation apparatus are increasingly used in cancer therapies. However, as chronic imbalances rather than loss of function mediate their pathogenesis, research models that allow for the study of the complex effects of drugs on tissue properties in proteostasis-associated diseases are almost completely lacking. Here, to determine the functional effects of impaired proteostatic fine-tuning, we applied a combination of materials science characterisation techniques to a cell-derived, *in vitro* model of bone-like tissue formation in which we pharmacologically perturbed protein degradation. We show that low-level inhibition of VCP/p97 and the proteasome, two major components of the degradation machinery, have remarkably different effects on the bone-like material that human bone-marrow derived mesenchymal stromal cells (hMSC) form *in vitro*. Specifically, whilst proteasome inhibition mildly enhances tissue formation, Raman spectroscopic, atomic force microscopy-based indentation, and electron microscopy imaging reveal that VCP/p97 inhibition induces the formation of bone-like tissue that is softer, contains less protein, appears to have more crystalline mineral, and may involve aberrant micro- and ultra-structural tissue organisation. These observations contrast with findings from conventional osteogenic assays that failed to identify any effect on mineralisation. Taken together, these data suggest that mild proteostatic impairment in hMSC alters the bone-like material they form in ways that could explain some pathologies associated with VCP/p97-related diseases. They also demonstrate the utility of quantitative materials science approaches for tackling long-standing questions in biology and medicine, and could form the basis for preclinical drug testing platforms to develop therapies for diseases stemming from perturbed proteostasis or for cancer therapies targeting protein degradation. Our findings may also have important implications for the field of tissue engineering, as the manufacture of cell-derived biomaterial scaffolds may need to consider proteostasis to effectively replicate native tissues.

## 1 Introduction

Accurate and stable maintenance of cellular protein homeostasis (proteostasis) is critical for tissue integrity and has been linked to longevity [[1], [2], [3], [4]]. Perturbed proteostasis contributes to the pathogenesis of a myriad of predominantly age-related diseases ranging from neurodegenerative disorders to diabetes and cancer [[5], [6], [7]]. An elaborate network of mechanisms constantly monitors and fine-tunes the intracellular proteome [8,9]. The controlled degradation of proteins that are dysfunctional, damaged, or no longer needed is central to this process and is primarily executed by the ubiquitin-proteasome system (UPS). The UPS recognises proteins that have been earmarked for degradation by the addition of polyubiquitin chains, and degrades them in the 26S proteasome, thereby regulating multiple cellular functions, including stem cell fate [[10], [11], [12], [13]]. Small molecule inhibitors of the proteasome are widely used in the treatment of multiple myeloma, and pharmacological targeting of other UPS components is a major area of anti-cancer research [14].

The ATPase VCP/p97 plays a central role in the UPS by extracting ubiquitinated proteins from cellular structures and delivering them to the proteasome [[15], [16], [17], [18], [19]]. Drugs targeting VCP/p97 have therapeutic potential as anti-cancer agents and for the treatment of epilepsy linked to GABAA receptor mutations [[20], [21], [22]]. VCP/p97 mutations have been associated with a multisystem degenerative disease that comprises Paget's disease of bone, inclusion body myopathy, and fronto-temporal dementia (IBMPFD), and with several other diseases of the nervous and muscular system [[23], [24], [25], [26], [27], [28]]. In short, VCP/p97 is essential for cellular proteostasis and maintains skeletal and neurological health [29].

Although the pathogenesis of VCP/p97-related diseases remains to be established, their associated cellular dysfunction has been linked to defective proteostatic fine-tuning [[29], [30], [31]]. This putative mechanism is compatible with the notion that relatively minor but chronic or intermittent imbalances in proteostasis contribute to many age-related diseases [4,29]. However, perturbations in fine-tuning that result in chronic or intermittent imbalances in intracellular proteostasis are extremely challenging to replicate in *in vitro* and particularly in *in vivo* models. Indeed, the relative difficulty of establishing a research model when impairment rather than loss of function mediates complex tissue pathologies has hampered research efforts to identify the pathogenesis of VCP/p97-related diseases. Moreover, there are currently no robust experimental paradigms to study the functional effects of intracellular proteostasis imbalance or test potential therapeutic compounds that modulate proteostasis. In short, a research platform that could mimic the functional tissue effects of chronic or intermittent proteostasis imbalances could be invaluable in both exploring disease mechanisms and screening for drug effects.

The bone-like material that can be formed by osteogenic cells *in vitro* constitutes a highly informative model system to study how impaired intracellular proteostasis might impact functional tissue properties. As mesenchymal stromal/stem cells (MSC) differentiate down the osteogenic lineage and synthesise large amounts of extracellular matrix (ECM), they become highly dependent on mechanisms which control proteostasis [[32], [33], [34]]. This secreted proteinaceous matrix is then progressively mineralised by poorly crystalline carbonated apatite, producing a bone-like nano-composite structure in a highly controlled process such that even small perturbations to the composition of either the proteinaceous or mineral phases can significantly impact bone quality [[35], [36], [37]], providing a read-out of proteostasis imbalance. This model is also of direct clinical relevance because the pathogenesis of VCP/p97-related bone disease is incompletely understood; and whilst proteasome inhibitors purportedly stimulate bone regeneration in myeloma patients, the effects of drugs targeting VCP/p97 on bone have not been established [[38], [39], [40]]. Moreover, cell-derived, ECM-based materials have been proposed as promising scaffolds to direct SC differentiation in tissue engineering applications [41,42]. Therefore, insight into how proteostasis imbalances may impact these biomaterials’ functional properties may be important for creating scaffolds that appropriately mimic native tissues.

To understand how impaired proteostatic fine-tuning functionally affected tissue, we created an *in vitro* model using intermittent low-level proteasome or VCP/p97 inhibition in human MSC (hMSC) as they differentiated into osteoblasts and formed a cell-derived, bone-like material (Supplementary Fig. 1). We show that low-level inhibition of VCP/p97 and the proteasome differentially affect the bone-like material that hMSC form *in vitro*. Indeed, whilst proteasome inhibition subtly promotes the formation of a material akin to native bone, Raman spectroscopic, atomic force microscopy (AFM)-based indentation, and electron microscopy imaging suggest that VCP/p97 inhibition results in a material that is softer and less proteinaceous, but whose mineral appears to be more crystalline and morphologically aberrant. These observations suggest that mild VCP/p97 impairment in hMSC undergoing osteogenic differentiation may alter tissue physical properties in a way that could explain some of the pathologies associated VCP/p97-related diseases, including changes in bone mechanical properties [43]. Our results highlight the utility of applying materials science approaches to challenges that biological techniques cannot yet address. They may also provide the basis for *in vitro* platforms that would allow for the functional effects of proteostasis imbalances to be evaluated quantitatively in a model that could be particularly relevant for high-throughput pre-clinical drug screening purposes. Finally, our findings suggest that the fabrication of biomaterial scaffolds that utilise cell-derived matrices may need to consider the effects of proteostasis in order to properly match scaffold properties to those of the native tissue.

## 2 Results

### 2.1 DBeQ and bortezomib induce a mild proteotoxic stress response in differentiating hMSC

**Fig. 1:**
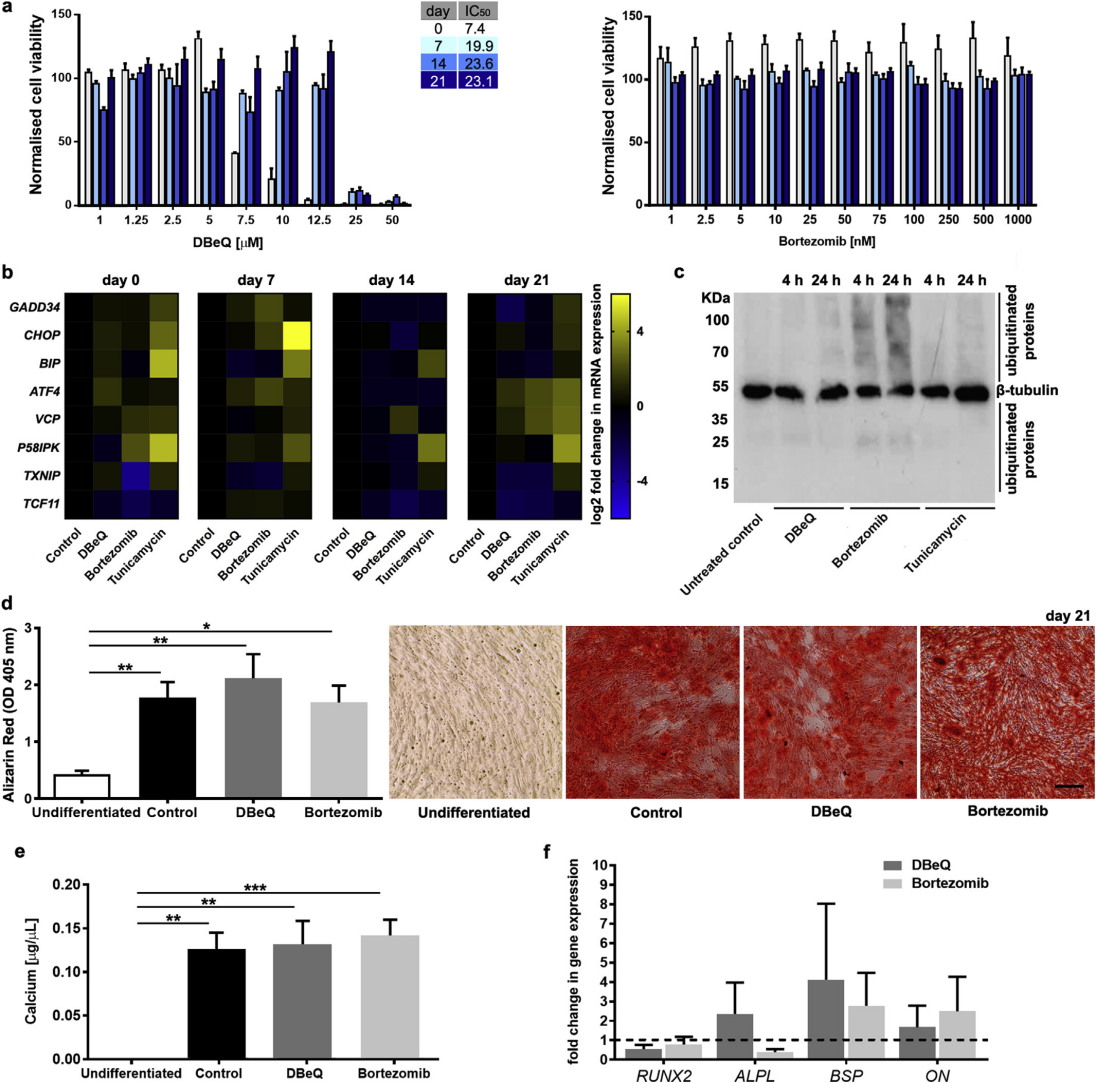
**Mild proteostasis perturbations do not grossly affect the osteogenic differentiation of hMSC**. **a**, Normalised viability of hMSC undergoing osteogenic differentiation treated on days 0, 7, 14, and 21 with the indicated concentrations of DBeQ or bortezomib for 48 h (*n* = 3). **b**, Heatmaps showing relative mRNA expression levels (normalised to undifferentiated controls) for *GADD34*, *CHOP, BIP*, *ATF4*, *VCP*, *P58IPK*, *TXNIP* and *TCF11* in differentiating hMSC on days 0, 7, 14, and 21 after treatment for 24 h with DBeQ (5 μM), bortezomib (20 nM), or tunicamycin (5 μg/mL). **c**, Immunoblotting for β-tubulin and ubiquitinated proteins on whole cell extracts from undifferentiated hMSC untreated (control) or treated for 4 h or 24 h with 5 μM DBeQ, 20 nM bortezomib, or 5 μg/mL tunicamycin. **d**, Quantification and representative micrographs showing Alizarin Red S staining (Scale bar = 200 μm) and **e**, colorimetric calcium content quantitation of differentiated hMSC cultures. **f**, Relative mRNA expression levels for markers of osteogenic differentiation compared to undifferentiated controls, which are set to 1 (dotted horizontal line). In **a**, **d**-**f**, plots show mean + SEM and in **b**, heat maps show mean of log 2 fold changes in gene expression (normalised to undifferentiated controls) for hMSC from 3 different donors. In **d**-**f**, a Kruskal-Wallis non-parametric test followed by Dunn's Multiple Comparison test was used to detect statistical significance, *p < 0.05, **p < 0.01 and ***p < 0.001. For detailed *n* and *p* values see Supplementary Tables 1-4. (For interpretation of the references to colour in this figure legend, the reader is referred to the Web version of this article.)

To develop an *in vitro* model of proteostasis imbalance, we first aimed to determine if we could mildly perturb proteostasis in hMSC undergoing osteogenic differentiation. Genetic approaches to deplete VCP/p97 or the proteasome are not suitable to study the effects of mild functional impairments [20,44]. Therefore, we took a pharmacological approach and treated hMSC with either the well-characterised and highly selective VCP/p97 inhibitor, DBeQ [[45], [46], [47], [48]], or the first-in-class clinical proteasome inhibitor, bortezomib [49]. To define inhibitor concentrations that would induce mild functional impairment without overt toxic effects, we initially determined IC_50_ values for viability. We found that osteogenic differentiation increased the IC_50_ for DBeQ (as determined by cellular metabolic activity) from 7.5 μM in undifferentiated hMSC to 22 μM in their differentiated progeny (Fig. 1a). For comparison, bortezomib, which effectively kills multiple myeloma cells *in vitro* at concentrations of 10–20 nM [47] (Supplementary Fig. 2), did not reduce viability of differentiating hMSC at concentrations up to 1000 nM (Fig. 1a). Next, we aimed to determine the degree of proteotoxic stress caused by a concentration of DBeQ that did not affect viability (5 μM) at any stage of *in vitro* differentiation compared to a clinically relevant concentration of bortezomib (20 nM) by quantifying the expression of a panel of genes encoding proteins with key roles in proteostasis. DBeQ and bortezomib both induced a very mild proteotoxic stress response, as determined by low-level changes in proteostasis gene mRNA levels that were largely non-significant (Fig. 1b and Supplementary Table 1). For comparison, the protein glycosylation inhibitor tunicamycin, which causes protein misfolding in the endoplasmic reticulum, resulted in more pronounced changes in proteostasis gene mRNAs when given at a nonlethal dose (Fig. 1b and Supplementary Table 1). However, immunoblotting for ubiquitinated proteins confirmed that bortezomib and DBeQ perturbed the UPS, with a clear increase in the level of ubiquitinated proteins in cells treated with bortezomib, while DBeQ had a minor effect (Fig. 1c and Supplementary Fig. 3). These observations demonstrate that by fine-tuning an appropriate dose in differentiating hMSC, DBeQ and bortezomib can impair intracellular proteostasis and trigger mild proteotoxic stress that is not acutely toxic to cells.

### 2.2 Neither VCP/p97 nor proteasome inhibition affect gross measures of hMSC osteogenic differentiation

Like primary osteoblasts, hMSC form bone-like mineralised nodules *in vitro* in response to chemical induction. To study the effects of VCP/p97 and proteasome inhibition on this process, we first used conventional Alizarin Red S staining (Fig. 1d and Supplementary Table 2) and colorimetric calcium content quantitation (Fig. 1e and Supplementary Table 3), and although subtle qualitative changes in staining patterns were sometimes evident, we found that neither DBeQ nor bortezomib treatment resulted in significant gross changes in the formation of mineralised nodules. We also quantified the expression of genes associated with osteogenic differentiation and found that mRNA levels of *RUNX2, ALPL, BSP*, *and ON* were not significantly affected by treatment with DBeQ or bortezomib (Fig. 1f, Supplementary Table 4 and Supplementary Fig. 4). Taken together, these data suggest that mild VCP/p97 or proteasome inhibition did not grossly affect the formation of bone-like mineralised nodules nor the expression of genes known to be key drivers of osteogenic differentiation *in vitro*.

### 2.3 Proteasome, but not VCP/p97 inhibition, subtly promotes mineralised matrix formation

**Fig. 2:**
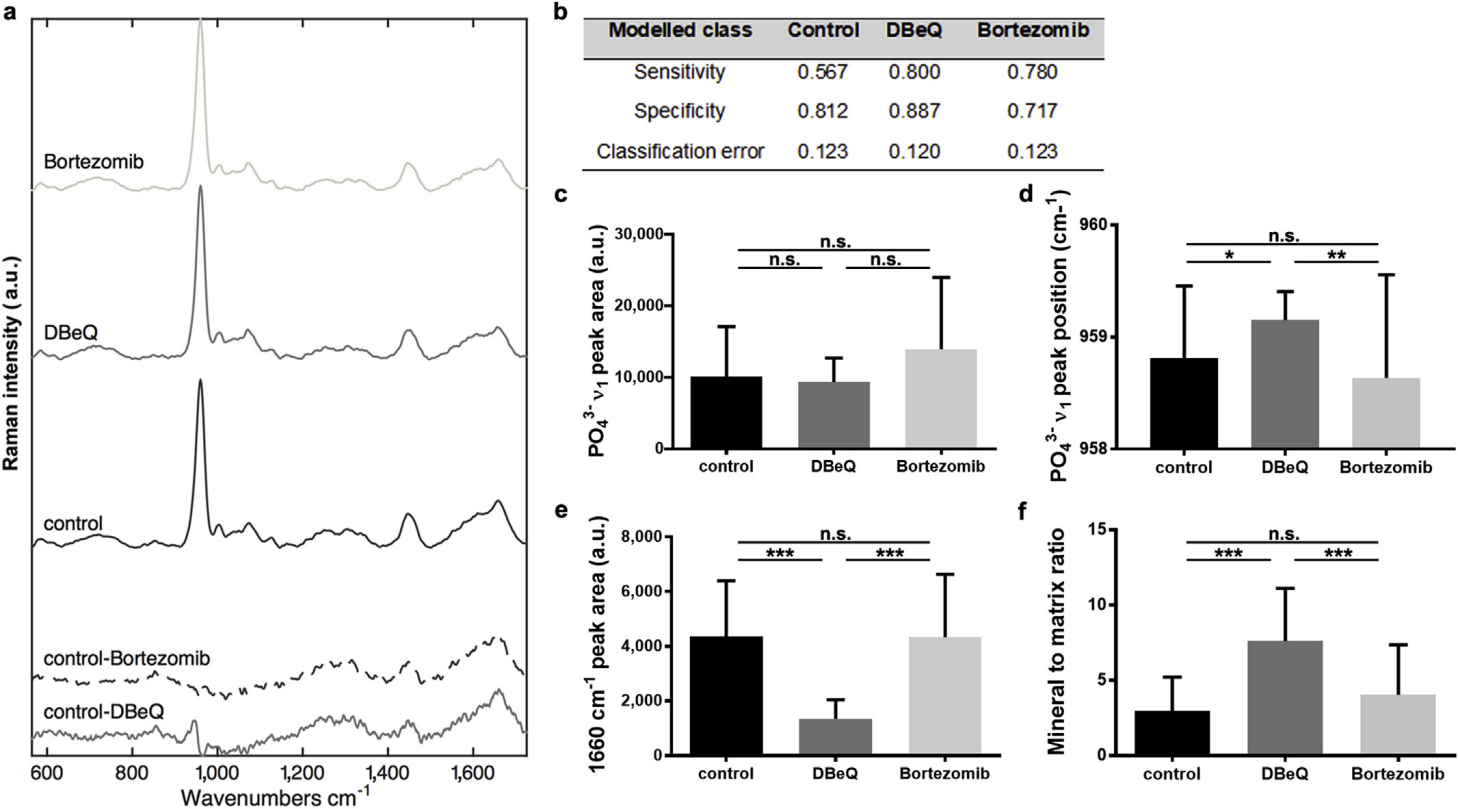
**VCP/p97 inhibition alters mineralised nodule composition as determined by Raman spectroscopy**. **a,** Mean Raman spectra collected from mineralised nodules formed in control, DBeQ- and bortezomib-treated hMSC cultures. Difference spectra of DBeQ- and bortezomib-treated cultures compared to controls are shown in the bottom of the panel. Spectra are offset on the y-axis for clarity. **b,** Table showing the fraction of spectra in DBeQ- and bortezomib-treated groups and the control that could be classified by sensitivity, specificity and classification error using a cross-validated 6-component Partial Least Squares-Discriminant Analysis model. **c,** Univariate analyses of the mean *PO*_*4*_^*3−*^ ν_1_ peak area at ∼960 cm^−1^ in control, DBeQ- and bortezomib-treated cultures, **d**, mean *PO*_*4*_^*3−*^ ν_1_ peak position, and **e**, 1660 cm^−1^ peak area. **f**, Mean mineral to matrix ratio in control, DBeQ- and bortezomib-treated cultures. In **c**,-**f**, data are means + SD and a Kruskal-Wallis non-parametric test followed by Dunn's Multiple Comparison test was used to detect statistical significance. *p < 0.05, **p < 0.01 and ***p < 0.001. For detailed *n* and *p* values see Supplementary Table 5.

Simple, gross observations of osteogenesis are qualitative and can fail to take into account more subtle aspects of bone formation and structure [50]. Therefore, to generate quantitative measures of the effects of proteostasis imbalances on tissue function, we next applied materials characterisation techniques to analyse the impact of VCP/p97 and proteasome inhibition on differentiating hMSC's ability to form a bone-like material *in vitro*. Raman spectroscopy detects vibrational chemical bonds, capturing the ‘biochemical signature’ of a substance. Raman spectroscopy has been used to characterise both the composition of native ECM [51] and that secreted by cultured cells [50,52,53]. Raman spectra collected from mineralised nodules formed under all conditions revealed a sharp peak at ∼960 cm^−1^ indicative of mineral phosphate ion vibrations (*PO*_*4*_^*3*−^ *ν*_*1*_) and other spectral features typical of native bone (Fig. 2a) [54]. This observation confirmed that all groups broadly formed a bone-like material, as previously described [50,52,53]. We then examined the integrated area of the *PO*_*4*_^*3*−^ *ν*_*1*_ peak, a relative measure of the amount of bone-like apatite [51]. Using standard univariate peak analysis, we observed an increase (although not statistically significant, *p* = 0.085) in the *ν*_*1*_ *PO*_*4*_^*3*−^ peak area in bortezomib-treated cultures compared to controls (Fig. 2c and Supplementary Table 5). VCP/p97 inhibition, on the other hand, did not alter the intensity of the *PO*_*4*_^*3*−^ *ν*_*1*_ peak. This suggests that whilst VCP/p97 inhibition did not affect the amount of mineral produced by hMSC, proteasome inhibition subtly enhanced it, a finding compatible with reports of the anabolic effects of proteasome inhibitors on bone [39,[55], [56], [57]], particularly in multiple myeloma [[58], [59], [60]].

### 2.4 VCP/p97, but not proteasome inhibition, impacts spectroscopic measures of mineral crystallinity and decreases proteinaceous matrix deposition

Raman spectral analyses not only provide information about the relative quantity of a substance, but also its structure. Therefore, we next analysed *PO*_*4*_^*3*−^ *ν*_*1*_ peak position as an indicator of mineral crystallinity. A downward shift is associated with a more amorphous, disorganised apatite whereas an upward shift suggests that the mineral is more crystalline [51]. Mean *ν*_*1*_ *PO*_*4*_^*3*−^ peak position for bortezomib-treated hMSC was similar to that of controls. However, in the DBeQ-treated group, it was significantly higher (Fig. 2d and Supplementary Table 5). A shift in *PO*_*4*_^*3*−^ *ν*_*1*_ peak position was also evident in difference spectra generated by comparing mean DBeQ- and bortezomib-treated conditions to controls (Fig. 2a, bottom-most spectra)). The ‘control-DBeQ’ spectrum produced a shift at ∼960 cm^−1^, whilst no shift was evident in the ‘control-bortezomib’ difference spectrum.

As VCP/p97 inhibition appeared to impact nodules’ mineral crystallinity, we next asked if the proteinaceous component had also been affected. The Raman peak centred at ∼1660 cm^−1^ has been attributed to Amide I and is an indication of protein content [51]. Bortezomib treatment did not affect the integrated area of the Amide I peak, however, it was significantly lower in DBeQ-treated nodules compared to controls (Fig. 2e and Supplementary Table 5), suggesting that VCP/p97 inhibition decreased the amount of Amide I-containing protein secreted by hMSC. This was reflected in mineral to matrix ratio, a measure of the relative amount of apatite to protein in nodules. Whilst bortezomib-treated nodules were no different, DBeQ treatment produced nodules with significantly higher mineral to matrix ratios compared to controls (Fig. 2f and Supplementary Table 5).

We next aimed to confirm the Raman spectroscopic distinctiveness of nodules formed when hMSC were subjected to VCP/p97 as opposed to proteasome inhibition by multi-variate analysis techniques. Using a 6-component Partial Least Squares-Discriminant Analysis model, we found that whilst all groups showed similar classification error, we were able to classify a larger fraction of DBeQ-treated spectra with better sensitivity and specificity than either control or bortezomib-treated groups (Fig. 2b). This suggests that whilst control and bortezomib-treated mineralised nodules were similar spectroscopically, DBeQ treatment produced spectra that were more unique and so amenable to classification in the model. Taken together, these observations suggest not only that VCP/p97 inhibition affected the composition of the cell-derived mineralised material created by differentiating hMSC, but also highlights that these effects were quantifiable using a relatively simple interdisciplinary technique.

### 2.5 VCP/p97, but not proteasome inhibition, decreases mineralised nodule stiffness

**Fig. 3:**
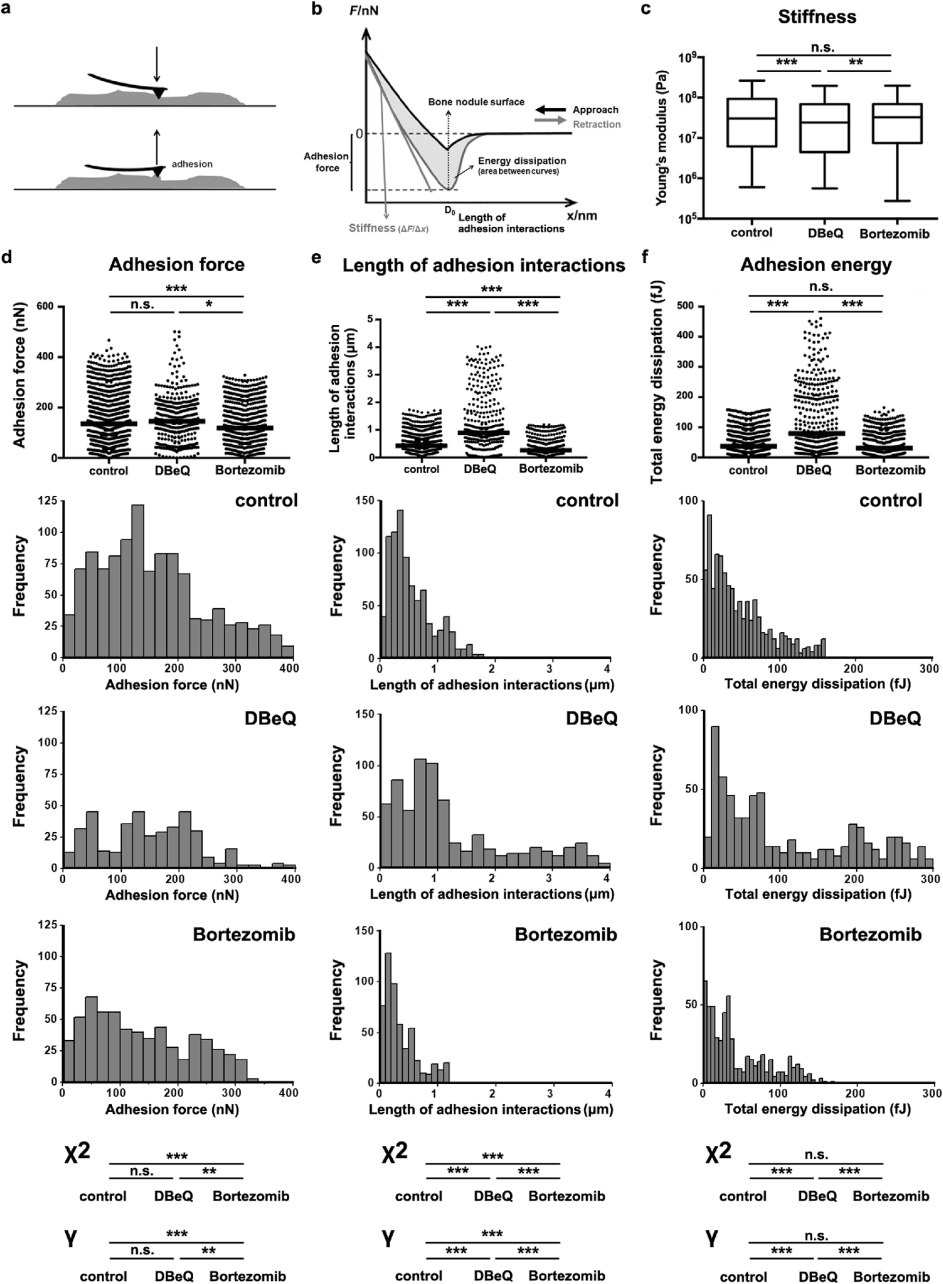
**Proteasome and VCP/p97 inhibition have differing effects on mineralised nodules' stiffness and adhesion interactions**. **a**, Schematic showing how mineralised nodules were probed by AFM in force-indentation mode using a cantilever with a pyramidal tip. **b**, Typical force-indentation curve generated from indenting a mineralised nodule. Schematic shows how nodule stiffness and adhesion interactions were calculated from the retraction curve. **c**, Measurements of Young's Modulus (Pa) of mineralised nodules. Plots show medians, 1st and 3rd quartiles and highest and lowest values. **d**, Measurements of adhesion force, **e**, length of adhesion interactions and **f**, adhesion energy generated from force-indentation curves on control, DBeQ- and bortezomib-treated cultures. Plots show medians. Histograms with their associated statistical analyses show how the distributions of values differed between the groups. In **c**.-**f**. a non-parametric Kruskal-Wallis test followed by Dunn's multiple comparison was used to determine statistical significance. Significant differences in the distributions of adhesion values were evaluated using a Mantel-Haenszel linear-by-linear association Chi-squared (*χ*^2^) test for trend. Power was evaluated by determining Goodman and Kruskal's gamma (γ). *p < 0.05, **p < 0.01 and ***p < 0.001. For detailed *n* and *p* values see Supplementary Table 6.

As VCP/p97 inhibition affected the biochemical composition of mineralised nodules, we next aimed to expand our *in vitro* model and determine its effect on nodules' nano-scale mechanical properties by measuring their stiffness. AFM is known for its high-resolution imaging capabilities; however, it is also a powerful tool for carrying out force-indentation measurements (Fig. 3a/b), producing quantitative insight into the mechanical properties of a material at the nano-scale [61]. We found that Young's modulus (*E*) was significantly lower in nodules treated with DBeQ compared to controls or those treated with bortezomib (Fig. 3c and Supplementary Table 6). As indentation was carried out with a probe whose tip radius was 8 nm (manufacturer's specification), this observation suggests that VCP/p97 inhibition significantly reduced the stiffness of the cell-derived material created by differentiating hMSC at the scale of the nano-composite structure of native bone.

### 2.6 Proteasome inhibition reduces the strength of adhesion interactions, but VCP/p97 inhibition enhances the total energy dissipated

In addition to probing their stiffness, AFM-based indentation measurements also reveal information regarding the cell-derived materials’ proteinaceous content. This is because upon indentation, the AFM probe forms non-specific adhesion interactions with the surface of the nodule (likely with proteins). During unloading of the indentation measurement, the deflection of the cantilever can reveal the strength of the adhesion interactions between the probe and the sample, as well as measure the distance over which those adhesion interactions take place. We first found that adhesion force (Fig. 3d, Supplementary Fig. 5, and Supplementary Table 6) was not different in DBeQ-treated samples compared to controls; however, in bortezomib-treated samples, it was significantly lower. This suggests that bortezomib-treated nodules interacted with the AFM probe less strongly, likely because the protein composition was different and/or was tightly bound to the more highly mineralised nodules and thus less available to interact with the AFM probe.

We next examined the length of the adhesion interactions, a measure of the distance over which adhesion interactions take place between the sample and the AFM probe during the unloading phase (Fig. 3e and Supplementary Table 6). DBeQ treatment produced interactions at significantly larger adhesion interactions lengths than those observed in either control or bortezomib-treated nodules. Analyses of the distributions showed that in DBeQ-treated nodules, interactions often took place as the probe retracted several microns from the contact point (2–4 μm), as confirmed by statistical analyses for trend (Fig. 3e and Supplementary Table 6). This contrasts with measurements on control and bortezomib-treated cultures, where most interactions took place within a few hundred nanometers of the contact point. This was also evident in measurements of total adhesion energy, which accounts for both adhesion strength and the distance over which it acts (Fig. 3f and Supplementary Table 6). All groups showed interactions with the probe at low values for total energy (first peak in histograms at < 20 fJ), likely reflecting interactions between the AFM probe and protein that was strongly bound to mineral. However, median adhesion energy was significantly higher in DBeQ-treated groups and was concomitant with many individual measurements at high values of energy dissipation. These observations suggest that VCP/p97 inhibition produced nodules that were highly compliant mechanically and whose protein may have been less tightly bound to the mineral (or the protein had different charge/adhesive properties), and thus could be extended away from the nodules and dissipate energy via interactions with the AFM probe. On the other hand, protein in control and bortezomib-treated nodules may have been more strongly bound to mineral, as in native bone. Moreover, these observations demonstrate that AFM, a widely-available technique, can identify quantifiable changes in tissue properties in response to mild VCP/p97 impairment.

### 2.7 VCP/p97, but not proteasome inhibition impacts the ultra- and micro-structure of mineralised nodules

**Fig. 4:**
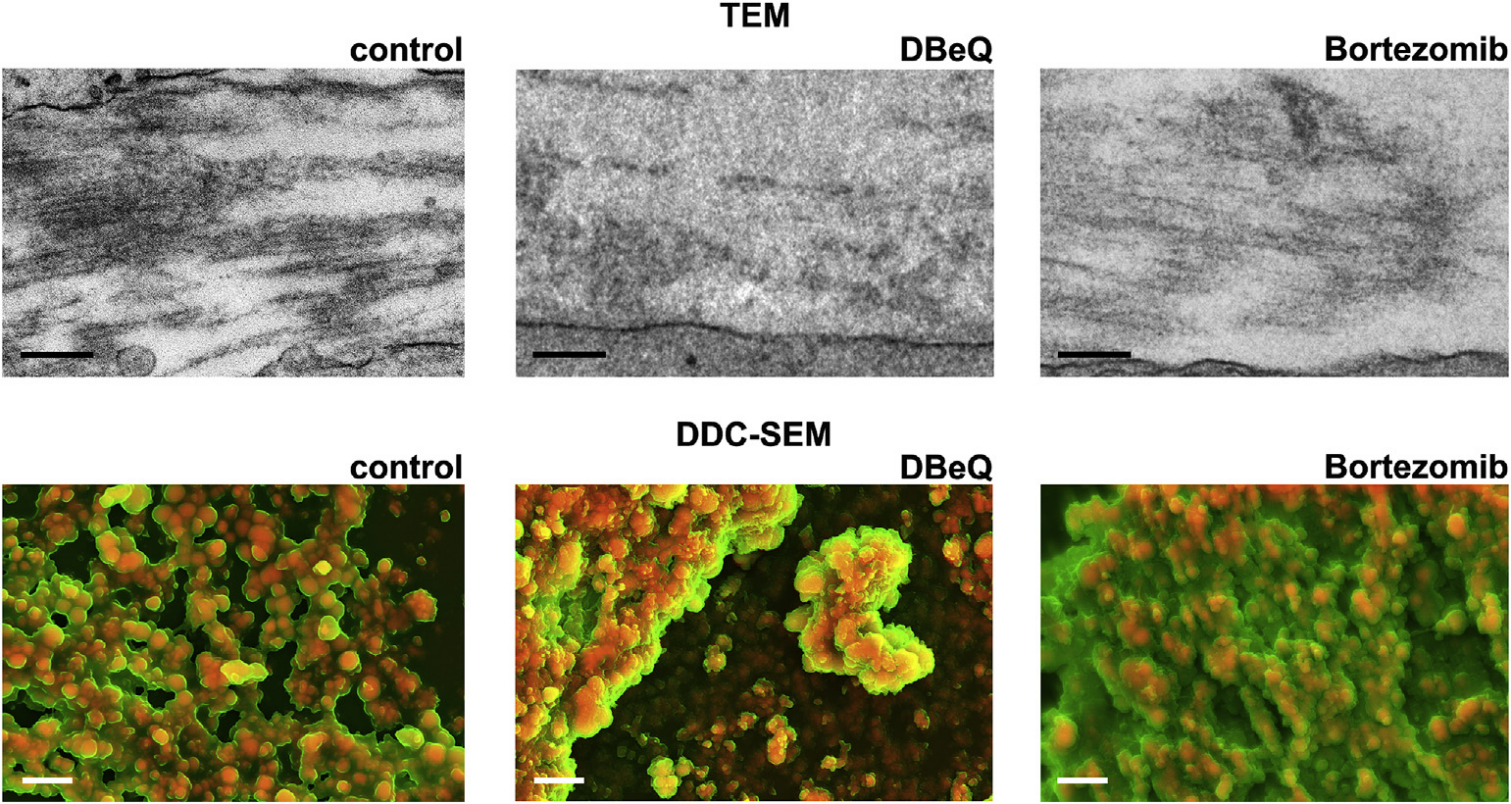
**VCP/p97 inhibition impacts the ultra- and micro-structure of mineralised nodules**. Transmission electron microscopy (TEM) and density-dependent colour scanning electron microscopy (DDC-SEM) micrographs of mineralised nodules formed from hMSC under standard osteogenic conditions or treated with DBeQ or bortezomib. In TEM images, proteinaceous fibrils are evident in the intercellular space in control and bortezomib-treated cultures. In DBeQ-treated samples, the proteinaceous matrix between cells appeared amorphous and clear fibrils were often not evident. Scale bar = 250 nm. In DDC-SEM micrographs, images were coloured in post-processing by combining images from secondary and backscatter electron detectors to identify dense mineral (red) and less dense matrix (green). In control and bortezomib-treated groups, there appears to be an association between the organic matrix and the mineral. In DBeQ-treated groups, dense mineral was often detected without the associated presence of less-dense matrix. Images are representative from experiments carried out on cultures grown from 3 independent donors. Scale bar = 2 μm. (For interpretation of the references to colour in this figure legend, the reader is referred to the Web version of this article.)

As VCP/p97 inhibition affected the biochemical composition, as well as the mechanical and adhesive properties of the bone-like material formed by hMSC, we next aimed to visualise its effects on this matrix using a combination of transmission (TEM) and scanning electron microscopy (SEM). TEM micrographs of control and bortezomib-treated cultures had similar ultrastructures to those previously described for bone-like nodules [50,62], and were notable for aligned fibrous protein between cells (Fig. 4). However, in DBeQ-treated cultures, we often observed more disorganised matrix in the intercellular space, and aligned fibrous protein was less common. We also examined nodules by SEM using both backscatter and secondary electron modes. Combining the two to create density-dependent colour SEM (DDC-SEM) images allowed us to visualise dense mineralised and less dense proteinaceous areas [63]. hMSC cultured under basal conditions did not form mineralised nodules (Supplementary Fig. 6). Images of the material formed under control conditions and those treated with bortezomib contained dense, mineralised areas that appeared to closely associate with less dense matrix, with morphologies similar to those previously described [64]. However, in DBeQ-treated cultures, although some dense material localised with matrix, we also observed highly dense areas with little to no associated less dense matrix. Moreover, the structure of the dense mineral in DBeQ-treated cultures appeared different morphologically. Whilst dense mineralised areas of control cultures appeared smooth, mineral in DBeQ-treated cultures appeared rougher, with needle-like crystals characteristic of non-physiological precipitation (Supplementary Fig. 7).

## 3 Discussion

Here, we used a combination of materials science-based characterisation approaches to show that very mild impairment of intracellular proteostasis can modify tissue physical properties in a cell-derived *in vitro* model that mimics the formation of native bone tissue. Alizarin Red S staining and calcium quantification showed that DBeQ- and bortezomib-treated as well as control cultures all produced a similar amount of mineralised material. However, by quantifying the *PO*_*4*_^*3*−^ *ν*_*1*_ peak intensity of Raman spectra, we determined that bortezomib-treated samples produced numerically (but not statistically significant) more apatite than other groups. Alizarin red S staining is a crude estimate of bone formation and whilst it detects the presence of calcium-containing compounds [41], bone mineral is more complex. Here, Raman spectral measurements were able to provide more specific information regarding bone-like tissue formation.

In our *in vitro* model, Raman spectroscopy identified quantitative effects of VCP/p97 inhibition, which contrasted with those of proteasome inhibition. Specifically, whilst DBeQ appeared to increase mineral crystallinity, bortezomib had no significant effect on spectroscopic measures of apatite structure. Mineral crystallinity increases with age [35] and has been associated with decreased bone ductility [36] and increased fracture risk [65], suggesting spectroscopic measurements may provide informative functional measurements of bone quality. VCP/p97 inhibition also significantly decreased nodule protein content, producing a higher mineral to matrix ratio when compared to controls. Although bortezomib treatment subtly promoted mineral formation, this was accompanied by appropriate formation of proteinaceous matrix, as mineral to matrix ratio was not different than that of controls. As bone formation is a highly controlled process in which mineral templates on a proteinaceous matrix, this observation suggests that whilst bortezomib increased mineralisation (in line with previous observations [39,57,58]), proteasome inhibition did not fundamentally interfere with this process. DBeQ treatment, on the other hand, induced the formation of mineral in the absence of appropriate proteinaceous matrix deposition, suggesting that the mineral may have been aberrant. As Raman spectral measurements are relatively straightforward and can be performed on almost any biological sample under any condition (wet/dry/fixed/unfixed/etc.) in the absence of labelling or staining, these observations highlight the technique's utility as a quantitative means to identify functional tissue effects of VCP/p97 perturbations.

One of bone's primary roles is to resist applied load. Therefore, its mechanical properties, including stiffness, are central to its function. Indeed, pathological changes in mechanical properties are associated with diseases including osteogenesis imperfecta, osteopetrosis, osteoporosis and rickets [66]. Our AFM-based indentation measurements showed that whilst bortezomib had no effect on the cell-derived materials' stiffness, DBeQ significantly decreased their Young's modulus. Differences in stiffness are likely not attributable to cultures forming less mineral, as both histochemical staining and spectroscopic analyses showed that VCP/p97 inhibition did not reduce the amount of mineral compared to control cultures. Instead, our observations suggest that DBeQ altered either the composition or structure of mineralised nodules. In bone, the protein-mineral nano-composite provides toughness via pre-stress within its structure. This is mediated by cross-linked collagen fibres, which impart compressive stress on mineral crystals as they form, whilst the growing mineral crystals tense the collagen structure [67]. Our observations that DBeQ-treated nodules were less stiff suggests that these composite interactions may have been limited.

Our AFM-based indentation measurements also produced highly informative observations with regards to adhesion interactions. Adhesion interactions with the AFM probe can be mediated by non-specific interactions, likely with protein via hydrophobic, ionic, steric, and/or Van der Waals forces [68]. We observed that DBeQ, but not bortezomib treatment, led to striking changes in key measures of adhesion, including energy dissipation, suggesting that protein within DBeQ-treated nodules was either less tightly bound to mineral or the proteome of DBeQ-treated nodules differed from that in control or bortezomib-treated groups. Bone mineralisation is thought to occur when negatively charged proteins stabilise disordered mineral precursors, which later become more crystalline and grow [69], creating a nano-composite structure. This structure requires a precise protein composition to allow for correct formation. Our observations suggest that in DBeQ-treated cultures, this balance may have been disrupted, precluding the formation of bone-like mineral with appropriate nanostructure. However, despite our quantitative insights, the effects of mild VCP/p97 impairment were subtle and not all adhesion interactions in DBeQ-treated groups displayed long-range, energy dissipative behaviours. Indeed, distributions revealed many measurements with short-range behaviour, similar to that in control and bortezomib-treated groups. Indeed, our observations suggest that either a subset of cells secreted protein that produced aberrant interactions, or a subset of the secreted proteome was aberrant.

TEM and SEM imaging suggested that DBeQ differentially affected the ultra- and micro-structure of the bone-like material created by hMSC compared to bortezomib treatment. Indeed, in DBeQ-treated samples, we observed amorphous rather than fibrillar proteinaceous matrix in the intercellular spaces. As apatite templates on fibrillar collagen in native bone, this observation lends further support to our AFM-based findings that mineral failed to strongly interact with appropriate protein to create a bone-like nano-structure. DDC-SEM images similarly suggested a lack of close association between less dense matrix and denser mineral in DBeQ-treated cultures. Moreover, high magnification secondary electron SEM images of DBeQ-treated cultures revealed needle-like mineral, akin to that observed in non-physiological precipitation reactions [70]. These observations suggest that in addition to an aberrant cell-mediated mineralisation process, the more crystalline mineral in DBeQ-treated cultures could have been formed in a pure physicochemical process, similar to that in pathological calcifications [63].

Here, we describe a cell-derived, biomaterial-based *in vitro* model of bone formation whereby we used pharmacological inhibition of two key components of the intracellular protein degradation apparatus to induce mild cellular stress. We then identified a series of relatively straightforward, widely available techniques from materials science that allowed us to quantitatively characterise changes to tissue biochemical composition, mechanical and adhesive properties, as well as observe morphological changes to their micro- and ultra-structure. This combination of materials science characterisation techniques is particularly amenable for analysing changes in bone-like tissue formation because neither AFM-based indentation, Raman spectroscopy nor the imaging techniques requires specific staining/labelling, which would require *a priori* knowledge of specific targets of the inhibition. The limitation of this approach, of course, is that specific targets are not identified; however, as VCP/p97 and the proteasome inhibitors affect the proteome broadly, this approach may be superior. Moreover, as the techniques analyse the physical material created by cells, they can also detect potential post-translational changes in proteins, which may impact on cell/tissue function. These changes may be missed by standard proteomic techniques, for example. In time, techniques such as single cell proteomics that can analyse post-translation modifications to proteins may become widely available; however, until then, physical science-based characterisations may remain the gold standard.

As our *in vitro* experimental platform allows for the functional effects of proteostasis imbalances to be evaluated quantitatively, it may find use in high-throughput pre-clinical drug screening purposes [71], particularly to evaluate the functional effects on tissues of therapies designed to treat diseases stemming from perturbed proteostasis or for cancer therapies targeting protein degradation. Given that multiple components of protein degradation pathways are being investigated as anti-cancer drug targets, our findings both highlight potential unwanted skeletal effects such approaches might bring, and also offer an early preclinical screening tool. Finally, cell-derived, ECM-based biomaterials akin to the mineralised nodules formed by our hMSC cultures are highly promising as potential scaffolds for tissue engineering [41,42]. As these materials are secreted/arranged by tissue-specific cells, they are both composed of appropriate ECM and contain tethered growth and other regulatory factors to direct seeded SC. However, tissue engineering approaches such as culture on 3D scaffolds can expose cells to reactive oxygen species, acidosis and hypoxia, all of which are known to perturb proteostasis and activate the unfolded protein response [72]. Our finding that mildly perturbing proteostasis significantly impacts tissue properties suggests that maintaining proteostasis may be essential in forming ECM-based biomaterial scaffolds that replicate the native tissue.

## 4 Conclusions

Using a cell-derived model of bone formation in which we mildly perturb proteostasis, we have shown that a combination of techniques from materials science can quantitatively detect tissue effects that may explain some of the pathologies associated with VCP/p97-related diseases. This model may have important implications for studying VCP/p97-related diseases. This is because there are no existing *in vivo* models that can replicate the subtle/intermittent proteostasis imbalances that characterise these diseases. Moreover, this is the first *in vitro* model we are aware of that examines the effects of proteostasis imbalances on the mechanical, morphological and biochemical characteristics of tissues, as previous *in vitro* studies using pharmacological agents to perturb proteostasis have only carried out limited molecular analyses, which provide little insight into the tissue pathologies observed in patients. Furthermore, whilst Raman spectroscopy, AFM and electron microscopy have been used to analyse tissues [51,63], including the material formed by cells *in vitro* [50,52,53]; to our knowledge this is the first report using these techniques to analyse tissue changes mediated by VCP/p97 and/or proteasome inhibition.

## 5 Methods

### 5.1 hMSC expansion

Human samples used in this research project were obtained from the Imperial College Healthcare Tissue Bank (ICHTB, HTA license 12275). ICHTB is supported by the National Institute for Health Research Biomedical Research Centre based at Imperial College Healthcare NHS Trust and Imperial College London. ICHTB is approved by the UK National Research Ethics Service to release human material for research (12/WA/0196), and the samples for this project were issued from sub-collection R16052. Bone marrow aspirates were obtained at St. Mary's Hospital Imperial College Healthcare NHS Trust (IHCNT) from healthy paediatric stem cell donors. hMSC were expanded for clinical use for the treatment of Graft-versus-Host-Disease. Written informed consent for the use of hMSC for research was obtained from the donors' parents.

Primary cultures were established using CellSTACK^®^ culture chambers, filling caps (Corning Incorporated, Life Sciences) and Macopharma seeding sets (Macopharma) with a grade A cleanroom environment under good manufacturing practice (GMP) conditions. We targeted a seed rate of 25 × 10^6^ total nucleated cells per level. Cells were cultured in Minimum Essential Medium α with GlutaMAX™ (αMEM, Gibco) supplemented with 5% in-house made platelet lysate. Platelets obtained by apheresis from 10 donors by the National Blood Service were pooled, centrifuged at 3000 rpm for 10 min to eliminate platelet bodies, and frozen at −80 °C. Platelet lysate pools were batch-tested for their ability to support hMSC growth. Cells were cultured in a humidified incubator in 5% CO_2_ at 37 °C until confluent. When confluent (usually after 14 days) cells were harvested and cryopreserved using a controlled rate freezer in 5% human albumin solution (Biotest) containing 10% DMSO (CryoPur™, OriGen) in a 1:1 ratio. Prior to cryopreservation, hMSC were immunophenotyped by staining with a panel of labelled mouse anti-human antibodies (CD105-APC, CD73-PE, CD90-APC, CD45-FITC, CD34-PE, CD3^−^, CD19-PE, HLA-DR-FITC and CD14-PE; BD Biosciences) using a FACSCalibur™ analyser (BD Biosciences) and CELLQUEST software. hMSC expressed CD90, CD105, and CD73 and were negative for hematopoietic markers CD34 and CD45 [73] (data not shown).

### 5.2 Cellular metabolic activity

Cellular metabolic activity as a read-out of cellular viability was measured using the Alamar Blue^®^ (Life Technologies) assay according to the manufacturer's instructions. The assay is based on the reduction of resazurin, a non-fluorescent dye, to highly fluorescent red resorufin in healthy cells. The Alamar Blue^®^ cell viability reagent was incubated at 10% of sample volume for 1–4 h at 37 °C, and the fluorescence (590 nm) of the sample measured using a FLUOstar Omega BMG Labtech plate reader. Viability was determined relative to untreated cells or cells treated with vehicle only.

### 5.3 Osteogenic differentiation of hMSC and inhibitors

hMSC were seeded at 5 × 10^3^ cells/cm^2^ in α-MEM. After 24 h, cells were approximately 80% confluent and osteogenic differentiation was initiated by switching to commercially available Stempro^®^ Osteogenesis Differentiation kit medium (Invitrogen) or osteogenic differentiation medium consisting of α-MEM, 15% FBS, 100 μM ascorbic acid (Scientific Lab Supplies, UK), 2.5 mM β-glycerophosphate (Calbiochem) and 100 nM dexamethasone (Sigma). For experiments on undifferentiated hMSC, cells were grown in α-MEM supplemented with 10% heat-inactivated foetal bovine serum (FBS, Sigma), 1000 U/mL penicillin, and 10 mg/mL streptomycin (Sigma). Medium was changed every 3–4 days, and cells were used until passage 8 only. VCP/p97 and the proteasome were inhibited with DBeQ (Biovision) or bortezomib (Calbiochem). Tunicamycin was purchased from Sigma.

### 5.4 Myeloma cell line

Human OPM-2 multiple myeloma cells (obtained from the Deutsche Sammlung von Mikroorganismen und Zellkulturen DSMZ; identity confirmed using short tandem repeat profiling of 10 loci (Core Genomic Facility, Medical School, University of Sheffield)) were cultured in RPMI-1640 (Invitrogen) supplemented with 10% FBS.

### 5.5 RNA extraction and reverse transcription

Cells were harvested and snap frozen using liquid nitrogen. RNA was extracted using the GeneJET RNA Purification kit (Thermo Scientific) followed by removal of genomic DNA according to the manufacturer's instructions. Remaining traces of DNA were removed using DNase I treatment (Invitrogen). cDNA synthesis was performed using the RevertAid First Strand cDNA Synthesis kit (Thermo Scientific) according to the manufacturer's instructions using an Applied Biosystems 2720 Thermal Cycler (Life Technologies).

### 5.6 Quantitation of gene expression by real-time PCR

PCR reactions were performed on an Applied Biosystems StepOnePlus™ PCR machine using 10 μL SYBR Green JumpStart™ Taq ready Mix™ PCR (Sigma), 0.48 μL sequence-specific primers at 300 nM (Supplementary Table 7) and 5 μL cDNA. A three-step cycle was employed: (1) denaturation at 94 °C for 2 min; (2) annealing/extension at 60 °C for 1 min; and (3) melting from 60 °C to 94 °C for 2.5 min. The ΔΔCT method was used to quantify fold changes in expression (2^−ΔΔCq^) of each gene of interest and normalised to the expression of *GAPDH* in undifferentiated control cells.

### 5.7 Immunobloting

Whole-cell protein extracts were prepared on ice using a lysis buffer (Cell Signalling) supplemented with Complete EDTA-free Protease Inhibitor Cocktail (Roche). Then, after 10 min on ice and centrifugation at 4 °C (14,000 rpm) for 10 min, the supernatant was collected. Protein concentration was measured using Bradford reagent (Bio-RAD) according to the manufacturer's instructions. Proteins were denatured at 100 °C for 5 min with standard SDS-PAGE Loading Buffer (200 mM Tris-Cl, pH 6.8), 400 mM DTT, 8% SDS, 0.4% bromophenol blue, 40% glycerol). Proteins were separated on a 10% SDS polyacrylamide gel using electrophoresis and transferred to PVDF membranes (GE Healthcare). Membranes were blocked with 0.1% TBST (Tris-Buffered Saline, Tween20) containing 5% non-fat milk for 1 h at room temperature, incubated with primary antibodies against ubiquitin (Cell Signalling Technology, cat. no. 39335) and beta-tubulin (Cell Signalling Technology, cat. no. 21465) and then with a secondary anti-rabbit IgG labelled with horseradish peroxidase (Cell Signalling Technology, cat. no. 70745). Finally, an electrochemiluminescence (ECL ™ Western Blotting Reagents, GE Healthcare) system was used to detect proteins.

### 5.8 Alizarin Red S staining

Mineralisation was measured by staining with Alizarin Red S (ARS), a Ca^2+^-binding dye. Cells were washed with phosphate-buffered saline (PBS; Sigma) and fixed in 10% formalin in PBS for 20 min. Cells were washed twice in PBS followed by staining with 2% (w/v) Alizarin Red S (Sigma) solution in dH_2_O (after adjusting the pH to 4.2 using 10% NH_4_OH) for 10 min at room temperature. The dye solution was drained and the cells washed with running water for 30 min. Cultures were visualised using an EVOS ×1 Core digital inverted cell imaging microscope system.

### 5.9 Calcium quantification

Differentiated cell cultures were rinsed with PBS and then incubated at 4 °C on an orbital shaker overnight in 0.5 M HCl. Calcium quantification was then performed on lysates using the Calcium Colorimetric Assay kit (BioVision) according to the manufacturer's instructions. Briefly, 10 μL of sample (HCl with dissolved nodules) or known standards were incubated with 90 μL of chromogenic reagent that binds to the complex formed between calcium ions and 0-cresolphthalein, and 60 μL of assay buffer. The absorbance was then measured at 575 nm using a FLUOstar Omega BMG Labtech plate reader. The calcium concentration in the samples was then calculated using a standard curve generated using serial dilutions (0–2 μg).

### 5.10 Raman spectroscopy measurements and analyses

For Raman spectroscopy measurements, hMSC were cultured as described above, but were seeded on MgF_2_ coverslips (Crystran, UK) instead of tissue culture plastic to facilitate Raman spectral analyses. MgF_2_ is a weak Raman scatterer, whilst tissue culture plastic produces an intense Raman signal [74]. After 21 days in culture, MgF_2_ coverslips were briefly rinsed with deionised H_2_0 and dried in a bell jar desiccator [50]. Raman measurements were performed using a custom build Raman system consisting of a Laser Quantum Ventus 532 nm Laser (Stockport, UK). The laser was coupled via free space optics into an Olympus BX60 microscope (Hamburg, DE). Raman scattered light was collected through a 50×/NA 0.8 objective and fibre coupled into an Acton SpectraPro 2500i f/6.5 spectrograph, using a 1200 lines/mm grating with a Princeton Instruments PIXIS 400F 1340 × 400 pixel CCD camera (Trenton, NJ) operating at −75 °C. Integration time was 10 s, averaged 3 times for each spectrum, using 30 mW at the sample. We have previously shown that these conditions do not cause spectral changes due to sample heating/burning [52]. For univariate peak analyses, between 295 and 315 spectra were examined per group.

Data were smoothed using a 5 point Savitzky-Golay filter and background corrected by a 5th order polynomial using an automated weighted least squares fitting method [75]. To determine the position of the *PO*_*4*_^*3−*^ ν_1_ peak at ∼960 cm^−1^, a single Gaussian was fit to the data, as previously described [76,77]. Gaussian fits were used to calculate the 960 cm^−1^ (*PO*_*4*_^*3−*^ ν_1_) and 1660 cm^−1^ (Amide I) peak areas. The mineral to matrix ratio was calculated by determining the ratio of the area of the 960 cm^−1^ peak to that of the 1660 cm^−1^ peak [50,51] after normalization using Multiplicative Scatter Correction. Spectra with a *PO*_*4*_^*3−*^ ν_1_ peak area of less than 500 were excluded from multivariate analyses and in total, spectra collected from 29 control-, 38 DBeQ- and 42 bortezomib-treated cultures were analysed. For classification analyses, a 6-component Partial Least Squares-Discriminant Analysis model was fitted and cross validated using venetian blinds and 10 splits.

### 5.11 Atomic force microscopy (AFM) measurements of nodule stiffness and adhesion

22 mm glass coverslips were prepared by soaking in FBS overnight and allowing to air dry. hMSC were seeded at 190,000 cells/coverslip. The next day, basal medium was replaced with osteogenic medium and cells were differentiated as described above. After 21 days, coverslips were frozen slowly [50]. Briefly, medium was aspirated from plates and replaced with a 1:1 solution of 20% DMSO in FBS:basal medium. Plates were then sealed with parafilm and placed within 2 nesting polystyrene boxes at −80 °C. Samples were defrosted at room temperature immediately prior to measurements. Coverslips were immobilised on 60 mm diameter tissue culture dishes (TPP, CH) by gluing small coverslips to the plate at the sample edges. Coverslips were immersed in PBS (without calcium and magnesium, pH 7.4, Gibco) and measurements were made at room temperature. Mineralised nodules were identified from bright field images as dense, opaque patches on the culture surface that varied in diameter between ∼35 and 125 μm. Indentation measurements were carried out on a JPK Nanowizard^®^ I AFM equipped with a cantilever (cantilever B, spring constant, K ∼16 N/m) with a pyramidal, silicon AFM probe (HQ:NSC35/Hard/AL BS, MikroMasch^®^, DE). Cantilevers were calibrated using the thermal method [78]. Analysis was performed using J Unicam and JPK SPM software 2.3 01/2006 (JPK Instruments AG, DE). Indentations were carried out at 1 Hz under a constant loading rate with a relative setpoint force set as 3650 nN and no dwelling time. The effect of the hard underlying substrate was assumed to be negligible as the indenter penetration was less than 10% of the nodule thickness [79,80]. Nanoindentations were made at between 1 and 5 locations on each nodule's surface. 20 individual force indentation curves were made at each location (1 × 1 μm^2^). Measurements were performed on at least 12 nodules per donor per treatment. Young's modulus (*E*), maximum adhesion force, maximum length of adhesion interactions, and adhesion energy were determined in JPK SPM software using the Hertz model. The Poisson's ratio of both the sample and tip were assumed to be 0.3 [81].

### 5.12 Transmission electron microscope (TEM) imaging

Differentiated hMSC cultures were scraped from plates and pelleted in PBS. Pellets were fixed with 1% (w/v) glutaraldehyde for 15 min, then washed in cacodylate buffer three times, followed by incubation in 1% (w/v) osmium tetroxide and 1.5% (w/v) potassium ferrocyanide in H_2_O for 1 h. Pellets were then washed in H_2_O three times and dehydrated in an ethanol/distilled water series at concentrations from 20 to 100% ethanol for 15 min in each. Samples were then infiltrated with Epon 812 (EMS) epoxy resin in absolute ethanol at ratios of 3:1, 2:1, 1:1, 1:2, 1:3 (ethanol:resin) and then 100% epoxy resin for 6 h each. Samples were then cured at 60 °C for 48 h. Resin blocks were sectioned on a Leica Ultracut EM FC7 ultramicrotome on an ultracut UC7 chassis into 80 nm sections, placed on grids, stained with Reynold's lead citrate for 15 min and 1% (w/v) uranyl acetate for 15 min, then imaged on a Jeol 1010 TEM at 80 KV.

### 5.13 Scanning electron microcope (SEM) imaging

Cultures in well plates were dehydrated using increasing concentrations of ethanol (20%, 30%, 40%, 50%, 60%, 70%, 80%, 90%, 100%, 100% and 100% for 10 min each) and mounted on SEM stubs using carbon tape. Silver painting and carbon coating was carried out using a Quorum K975X Carbon coater. Samples were imaged on either a Hitatchi S—3499N or a Zeiss Sigma. For DDC-SEM images, secondary electron (SE) mode was used to obtain topographic information, whilst the backscattered electron detector (BSE) was applied to differentiate between organic and inorganic material. DDC-SEM images were produced by assigning different colours to the BSE and SE modes using Image J.

### 5.14 Statistical analyses

Statistical analyses for all measurements were carried out using a non-parametric Kruskal-Wallis test followed by Dunn's multiple comparison test unless stated otherwise. All analyses were carried out using GraphPad Prism version 7 for Windows (GraphPad Software, USA). Significant differences in the distributions of adhesion values between treatments were evaluated using a Mantel-Haenszel linear-by-linear association Chi-squared (*χ*^2^) test for trend. Power was evaluated by determining Goodman and Kruskal's gamma (γ) and standardised residuals (SR) were used to identify the most significant intervals that contributed to differences between histograms, all three using IBM^®^ SPSS^®^ statistics version V23. *p* values are indicated in figure captions, *p < 0.05, **p < 0.01 and ***p < 0.001 and showed in detail in supplementary tables.

## Author contributions

EG and HWA conceived the study, supervised the project, analysed data, and wrote the manuscript. SL, SAF, TMC, AK, ET, CD, KP, APS, SB, LB and MABH conducted experiments, analysed data, and contributed to writing the manuscript.

## Conflicts of interest

HWA has received research support unrelated to this project from Amgen, and honoraria from Novartis, Amgen, and Karyopharm.

## Data availability

The raw/processed data required to reproduce these findings cannot be shared at this time due to technical or time limitations.
