# Supplementary material for: An engineered, quantifiable in vitro model for analysing the effect of proteostasis-targeting drugs on tissue physical properties
Source: Biomaterials. 2018 Nov;183:102–13. doi: 10.1016/j.biomaterials.2018.08.041 (PMC6145445; doi:10.1016/j.biomaterials.2018.08.041)
Supplement: Supplementary file 1 [file mmc1.docx]

**Supplementary Information**

**Supplementary Tables**

**Supplementary Table 1**: mRNA expression analysis of hMSC (*n* = 3) treated on days 0, 7, 14, or 21 of osteogenic differentiation with DBeQ (5 μM), bortezomib (20 nM), or tunicamycin (5 μg/mL) for 24h. Results shown are log_2_ fold changes in mRNA expression compared to vehicle-treated controls and correspond to those shown in Figure 1b heatmaps. Statistical analyses were carried out using a two-way ANOVA and Dunnett’s multiple comparisons test. Significant results (p < 0.05) are highlighted in red.

|  | **DBeQ** | | | | **bortezomib** | | | | **tunicamycin** | | | |
| --- | --- | --- | --- | --- | --- | --- | --- | --- | --- | --- | --- | --- |
|  | **mean** | **±** | **SEM** | ***p*** | **mean** | **±** | **SEM** | ***p*** | **mean** | **±** | **SEM** | ***p*** |
| **day 0** | | | | | | | | | | | | |
| ***GADD34*** | 0.534 | ± | 0.356 | 0.937 | 0.535 | ± | 0.369 | 0.937 | 1.409 | ± | 1.101 | 0.469 |
| ***CHOP*** | 0.273 | ± | 0.631 | 0.991 | -0.090 | ± | 0.785 | 1.000 | 3.249 | ± | 1.344 | 0.016 |
| ***BIP*** | -0.229 | ± | 1.105 | 0.994 | -0.248 | ± | 0.165 | 0.993 | 4.622 | ± | 0.800 | 0.000 |
| ***ATF4*** | -0.169 | ± | 1.632 | 0.998 | -0.037 | ± | 0.649 | 1.000 | 1.424 | ± | 0.892 | 0.460 |
| ***VCP*** | 0.097 | ± | 0.237 | 1.000 | 1.280 | ± | 0.216 | 0.543 | 1.250 | ± | 0.555 | 0.561 |
| ***P58IPK*** | -0.196 | ± | 0.791 | 0.997 | 1.776 | ± | 0.733 | 0.286 | 4.015 | ± | 1.625 | 0.002 |
| ***TXNIP*** | -0.737 | ± | 1.524 | 0.856 | -2.822 | ± | 0.947 | 0.043 | 0.844 | ± | 1.152 | 0.801 |
| ***TCF11*** | -1.148 | ± | 0.855 | 0.623 | -2.118 | ± | 0.131 | 0.166 | -0.816 | ± | 1.126 | 0.816 |
| **day 7** | | | | | | | | | | | | |
| ***GADD34*** | 0.161 | ± | 1.076 | 0.999 | 1.724 | ± | 0.375 | 0.635 | 1.012 | ± | 0.881 | 0.889 |
| ***CHOP*** | 1.261 | ± | 1.339 | 0.811 | 3.125 | ± | 1.879 | 0.187 | 5.902 | ± | 1.849 | 0.004 |
| ***BIP*** | -1.319 | ± | 1.309 | 0.791 | -0.325 | ± | 1.076 | 0.995 | 3.722 | ± | 0.903 | 0.093 |
| ***ATF4*** | 0.952 | ± | 1.648 | 0.905 | 2.560 | ± | 1.210 | 0.330 | 2.374 | ± | 1.559 | 0.390 |
| ***VCP*** | -0.172 | ± | 0.231 | 0.999 | 0.778 | ± | 0.820 | 0.945 | 0.925 | ± | 0.843 | 0.912 |
| ***P58IPK*** | 1.353 | ± | 1.224 | 0.778 | 1.350 | ± | 2.336 | 0.780 | 3.455 | ± | 2.164 | 0.129 |
| ***TXNIP*** | -1.323 | ± | 0.450 | 0.789 | -1.090 | ± | 0.578 | 0.867 | 0.640 | ± | 0.892 | 0.976 |
| ***TCF11*** | -1.750 | ± | 2.778 | 0.624 | -0.252 | ± | 1.161 | 0.998 | -1.090 | ± | 1.951 | 0.867 |
| **day 14** | | | | | | | | | | | | |
| ***GADD34*** | -2.036 | ± | 1.180 | 0.635 | -1.275 | ± | 2.232 | 0.870 | -1.706 | ± | 1.434 | 0.745 |
| ***CHOP*** | -2.569 | ± | 3.781 | 0.460 | -2.323 | ± | 3.612 | 0.539 | -0.743 | ± | 1.876 | 0.969 |
| ***BIP*** | -0.679 | ± | 0.243 | 0.976 | -1.152 | ± | 0.871 | 0.899 | 2.872 | ± | 1.000 | 0.371 |
| ***ATF4*** | -2.036 | ± | 1.180 | 0.635 | -1.275 | ± | 2.232 | 0.870 | -1.706 | ± | 1.434 | 0.745 |
| ***VCP*** | -0.279 | ± | 1.021 | 0.998 | 1.239 | ± | 1.162 | 0.879 | 0.508 | ± | 1.141 | 0.990 |
| ***P58IPK*** | -0.749 | ± | 0.620 | 0.968 | -0.001 | ± | 1.939 | 1.000 | 2.289 | ± | 0.894 | 0.550 |
| ***TXNIP*** | 0.463 | ± | 1.287 | 0.992 | -0.330 | ± | 0.894 | 0.997 | 1.633 | ± | 1.239 | 0.767 |
| ***TCF11*** | -0.799 | ± | 1.397 | 0.962 | -1.363 | ± | 1.125 | 0.847 | -1.112 | ± | 1.575 | 0.908 |

|  | **DBeQ** | | | | **bortezomib** | | | | **tunicamycin** | | | |
| --- | --- | --- | --- | --- | --- | --- | --- | --- | --- | --- | --- | --- |
|  | **mean** | **±** | **SEM** | ***p*** | **mean** | **±** | **SEM** | ***p*** | **mean** | **±** | **SEM** | ***p*** |
| **day 21** | | | | | | | | | | | | |
| ***GADD34*** | -1.980 | ± | 1.163 | 0.369 | -0.174 | ± | 0.969 | 0.999 | 0.942 | ± | 1.006 | 0.845 |
| ***CHOP*** | -0.503 | ± | 0.998 | 0.971 | -0.846 | ± | 0.532 | 0.881 | 1.137 | ± | 1.034 | 0.761 |
| ***BIP*** | -0.367 | ± | 0.565 | 0.988 | -1.166 | ± | 0.685 | 0.748 | 1.273 | ± | 2.425 | 0.697 |
| ***ATF4*** | 1.582 | ± | 0.438 | 0.546 | 1.168 | ± | 1.026 | 0.747 | -0.262 | ± | 3.281 | 0.996 |
| ***VCP*** | 0.779 | ± | 0.392 | 0.904 | 1.856 | ± | 0.259 | 0.420 | 2.689 | ± | 0.386 | 0.152 |
| ***P58IPK*** | 0.181 | ± | 1.056 | 0.999 | 0.509 | ± | 1.130 | 0.970 | 3.348 | ± | 0.852 | 0.055 |
| ***TXNIP*** | -0.692 | ± | 1.220 | 0.929 | -1.681 | ± | 0.222 | 0.500 | 1.266 | ± | 0.918 | 0.701 |
| ***TCF11*** | -1.379 | ± | 1.001 | 0.645 | -1.869 | ± | 0.287 | 0.415 | -0.678 | ± | 0.897 | 0.933 |

|  |  |  |  |
| --- | --- | --- | --- |

**Supplementary Table 2**: Statistical analyses of Alizarin Red S staining quantification in undifferentiated hMSC cultured for 21 days in medium without osteogenic agents and hMSC undergoing differentiation with DBeQ or bortezomib treatment on days 7, 10, 14 and 17 or vehicle control.

| **Alizarin Red S staining quantification** | |
| --- | --- |
| Undifferentiated vs. control | *p* = 0.003 |
| Undifferentiated vs. DBeQ | *p* = 0.001 |
| Undifferentiated vs. bortezomib | *p* = 0.013 |
| control vs. DBeQ | *p* > 0.999 |
| control vs. bortezomib | *p* > 0.999 |
| DBeQ vs. bortezomib | *p* > 0.999 |

Statistical analyses were carried out using a non-parametric Kruskal-Wallis test followed by Dunn's multiple comparison test. *n* = 9 from 3 different donors.

**Supplementary Table 3**: Statistical analyses of colorimetric calcium content quantitation in undifferentiated hMSC cultured for 21 days in medium without osteogenic agents and hMSC undergoing differentiation with DBeQ or bortezomib treatment on days 7, 10, 14 and 17 or vehicle control.

| **Colorimetric calcium content quantitation** | |
| --- | --- |
| Undifferentiated vs. control | *p* = 0.002 |
| Undifferentiated vs. DBeQ | *p* = 0.003 |
| Undifferentiated vs. bortezomib | *p* = 0.001 |
| control vs. DBeQ | *p* > 0.999 |
| control vs. bortezomib | *p* > 0.999 |
| DBeQ vs. bortezomib | *p* > 0.999 |

Statistical analyses were carried out using a non-parametric Kruskal-Wallis test followed by Dunn's multiple comparison test. *n* = 9, from 3 different donors.

**Supplementary Table 4**: Statistical analyses of fold changes in osteogenic gene mRNA levels. hMSC undergoing osteogenic differentiation were treated with DBeQ, bortezomib or vehicle (control) on days 7, 10,14 and 17 and mRNA was extracted on day 21.

|  | **control vs. DBeQ** | **control vs. bortezomib** | **DBeQ vs. bortezomib** |
| --- | --- | --- | --- |
| ***RUNX2*** | *p* = 0.286 | *p* = 0.866 | *p* > 0.999 |
| ***ALPL*** | *p* > 0.999 | *p* = 0.388 | *p* = 0.286 |
| ***BSP*** | *p* > 0.999 | *p* > 0.999 | *p* > 0.999 |
| ***ON*** | *p* > 0.999 | *p* > 0.999 | *p* > 0.999 |

Statistical analyses were carried out using a non-parametric Kruskal-Wallis test followed by Dunn's multiple comparison test. *n* = 3, from 3 different donors.

**Supplementary Table 5**: Statistical analyses of univariate analyses of the mean *PO_4_^3-^ ν_1_* peak area at ~960 cm^-1^, mean *PO_4_^3-^ ν_1_* peak position, 1660 cm^-1^ peak area and mean mineral to matrix ratio in control, DBeQ- and bortezomib-treated cultures.

| **PO_4_^3-^ ν_1_ peak area** | | |
| --- | --- | --- |
| control (*n* = 70) vs.  DBeQ (*n* = 62) | control (*n* = 70) vs. bortezomib (*n* = 85) | DBeQ (*n* = 62) vs. bortezomib (*n* = 85) |
| K-W, D: *p* > 0.999 | K-W, D: *p* = 0.085 | K-W, D: *p* = 0.150 |
| **PO_4_^3-^ ν_1_ peak position** | | |
| control (*n* = 80) vs.  DBeQ (*n* = 64) | control (*n* = 80) vs. bortezomib (*n* = 86) | DBeQ (*n* = 64) vs. bortezomib (*n* = 86) |
| K-W, D: *p* = 0.013 | K-W, D: *p* > 0.999 | K-W, D: *p* = 0.005 |
| **1660 cm^-1^ peak area** | | |
| control (*n* = 76) vs.  DBeQ (*n* = 64) | control (*n* = 76) vs. bortezomib (*n* = 79) | DBeQ (*n* = 64) vs. bortezomib (*n* = 79) |
| K-W, D: *p* < 0.001 | K-W, D: *p* > 0.999 | K-W, D: *p* < 0.001 |
| **Mineral to matrix ratio** | | |
| control (*n* = 78) vs.  DBeQ (*n* = 59) | control (*n* = 78) vs. bortezomib (*n* = 80) | DBeQ (*n* = 59) vs. bortezomib (*n* = 80) |
| K-W, D: *p* < 0.001 | K-W, D: *p* = 0.158 | K-W, D: *p* < 0.001 |

Statistical analyses were carried out using a non-parametric Kruskal-Wallis (K-W) test followed by Dunn's (D) multiple comparison test. All *n* values were obtained after excluding outliers using a ROUT test.

**Supplementary Table 6**: Statistical analyses of distributions of stiffness (Young’s modulus), adhesion force, adhesion position, and adhesion energy values obtained by probing mineralised nodules by AFM.

| **Young’s modulus** | | |
| --- | --- | --- |
| control (*n* = 2090) vs. DBeQ (*n* = 1831) | control (*n* = 2090) vs. bortezomib (*n* = 1516) | DBeQ (*n* = 1831) vs. bortezomib (*n* = 1516) |
| K-W, D: *p* < 0.001 | K-W, D: *p* = 0.068 | K-W, D: *p* = 0.003 |
| **Adhesion force** | | |
| control (*n* = 1102) vs. DBeQ (*n* = 411) | control (*n* = 1102) vs. bortezomib (*n* = 613) | DBeQ (*n* = 411) vs. bortezomib (*n* = 613) |
| K-W, D: *p* > 0.999  χ^2^ = 0.580, *p* = 0.446  γ = -0.007, *p* = 0.842  *SR* (N): none | K-W, D: *p* < 0.001  χ^2^ = 20.690, *p* < 0.001  γ = -0.129, *p* < 0.001  *SR* (N): <2x10^-8^,  1.2x10^-7^—1.4x10^-7^, 1.8x10^-7^—2x10^-7^,  2.2x10^-7^—2.6x10^-7^, 3.2x10^-7^—3.8x10^-7^ | K-W, D: *p* = 0.012  χ^2^ = 9.121, *p* = 0.003  γ = -0.104, *p* = 0.007  *SR* (N): 6x10^-8^—1x10^-7^, 1.2x10^-7^—1.4x10^-7^, 1.8x10^-7^—2.2x10^-7^, 2.4x10^-7^—2.6x10^-7^ |
| **Length of adhesion interactions** | | |
| control (*n* = 887) vs. DBeQ (*n* = 360) | control (*n* = 887) vs. bortezomib (*n* = 541) | DBeQ (*n* = 360) vs. bortezomib (*n* = 541) |
| K-W, D: *p* < 0.001  χ^2^ = 254.559, *p* < 0.001  γ = 0.509, *p* < 0.001  *SR*  (m): <2x10^-7^—6x10^-7^, 1x10^-6^—1.2x10^-6^,  1.8x10^-6^—3.6x10^-6^ | K-W, D: *p* < 0.001  χ^2^ = 78.046, *p* < 0.001  γ = -0.352, *p* < 0.001  *SR*  (m): <2x10^-7^, 6x10^-7^,  7—8x10^-7^,  1.2x10^-6^—1.6x10^-6^ | K-W, D: *p* < 0.001  χ^2^ = 257.383, *p* < 0.001  γ = -0.706, *p* < 0.001  *SR*  (m): <2x10^-7^—1x10^-6^, 1.2x10^-6^—3.6x10^-6^ |
| **Adhesion energy** | | |
| control (*n* = 858) vs. DBeQ (*n* = 369) | control (*n* = 2090) vs. bortezomib (*n* = 558) | DBeQ (*n* = 369) vs. bortezomib (*n* = 558) |
| K-W, D: *p* < 0.001  χ^2^ = 279.778, *p* < 0.001  γ = 0.487, *p* < 0.001  *SR* (J): <2x10^-14^—6x10^-14^, 1.6x10^-13^—3x10^-13^ | K-W, D: *p* = 0.223  χ^2^ = 1.692, *p* = 0.193  γ = -0.072, *p* = 0.064  *SR* (J): 4x10^-14^—6x10^-14^, 1.4x10^-13^—1.6x10^-13^ | K-W, D: *p* < 0.001  χ^2^ = 213.847, *p* < 0.001  γ = -0.525, *p* < 0.001  *SR* (J): <2x10^-14^—4x10^-14^, 1.8x10^-13^—3x10^-13^ |

Statistical analyses were carried out using a non-parametric Kruskal-Wallis (K-W) test followed by Dunn's (D) multiple comparison test. A Mantel-Haenszel linear-by-linear association Chi-square test for trend (χ^2^) (degrees of freedom = 1) was used to test for whether the distributions were significantly different from one another. A non-parametric Goodman and Kruskal's gamma (γ) test was then applied to measure of the strength of association that exists between any two comparisons. Low values for association indicate that two distributions are highly similar, while higher values are evidence of a stronger association and indicate that the distributions are different: none (0.00 – ± 0.01), weak association (± 0.01 – 0.09), moderate association (± 0.10 – 0.29), evident strong association (± 0.30 – 0.99). Standardised residuals (*SR*) highlight the most significant areas of the histograms that contributed to differences. All *n* values were obtained after excluding outliers using a ROUT test.

**Supplementary Table 7**: Primers used for gene expression analyses.

| **Ubiquitin-proteasome system** | | | |
| --- | --- | --- | --- |
| Activating transcription factor 4 | *Atf4* | F | 5’-AGATGACCTGGAAACCATGC-3’ |
|  |  | R | 5’-AGGGATCATGGCAACGTAAG-3’ |
|  | | | |
| Binding immunoglobulin protein | *Bip* | F | 5’-GCCGTCCTATGTCGCCTTC-3’ |
|  |  | R | 5’-TGGCGTCAAAGACCGTGTTC-3’ |
|  | | | |
| C/EBP homologous protein | *Chop* | F | 5’-AGCAGAGGTCACAAGCACCT-3’ |
|  |  | R | 5’-TTCATGCTTGGTGCAGATTC-3’ |
|  | | | |
| Growth arrest and DNA damage-inducible protein | *Gadd34* | F | 5’-CTGAGCCCTGCCCCTTCCGAR-3’ |
|  |  | R | 5’- GAAGCGCACCTTTCTGGCCT-3’ |
|  | | | |
| CDK11 (p58) protein kinase | *p58* | F | 5’-TCCTGGTGGACCTGCAGTACG-3’ |
|  |  | R | 5’-CTGCGAGTAATTTCTTCCCC-3’ |
|  | | | |
| P97/ valosin containing protein (VCP) | *p97* | F | 5’-CCCTGTGCCTGCTTCTTT-3’ |
|  |  | R | 5’-GCTGCTCCCTTTCCACCA-3’ |
|  | | | |
| Transcription factor 11 | *Tcf11* | F | 5’-GGAGGATTTGGGGGCTGTAG-3’ |
|  |  | R | 5’-TCCTGTGCCAAAGGATGTC-3’ |
|  | | | |
| Thioredoxin-interacting protein | *Txnip* | F | 5’-TTCGGGTTCAGAAGATCAGG-3’ |
|  |  | R | 5’-TGGATCCAGGAACGCTAAC-3’ |
| **Osteogenic genes** | | | |
| Runt-related transcription factor 2 | *RUNX2* | F | 5’-ACAGTAGATGGACCTCGGGA-3’ |
|  |  | R | 5’-ATACTGGGATGAGGAAATGC-3’ |
|  | | | |
| Alkaline Phosphatase | *ALPL* | F | 5’-AACATCAGGGACATTGACGTG-3’ |
|  |  | R | 5’-GTATCTCGGTTTGAAGCTCTTCC-3’ |
|  | | | |
| Bone sialoprotein | *BSP* | F | 5’-GGGCAGTAGTGACTCATCCG-3’ |
|  |  | R | 5’-TCAGCCTCAGAGTCTTCATCTTC-3’ |
|  | | | |
| Osteonectin | *ON* | F | 5’-GATGGTGCAGAGGAAACCGA-3’ |
|  |  | R | 5’- TTTGCAAGGCCCGATGTAGT-3’ |
|  | | | |
| **Reference gene** | | | |
| Glyceraldehyde 3-phosphate dehydrogenase | *GAPDH* | F | 5’-TCTGCTCCTCCTGTTCGACA-3’ |
|  |  | R | 5’-AAAGCAGCCCTGGTGACC-3’ |

Primers were obtained from Integrated DNA Technologies.

**Supplementary Figures**

**
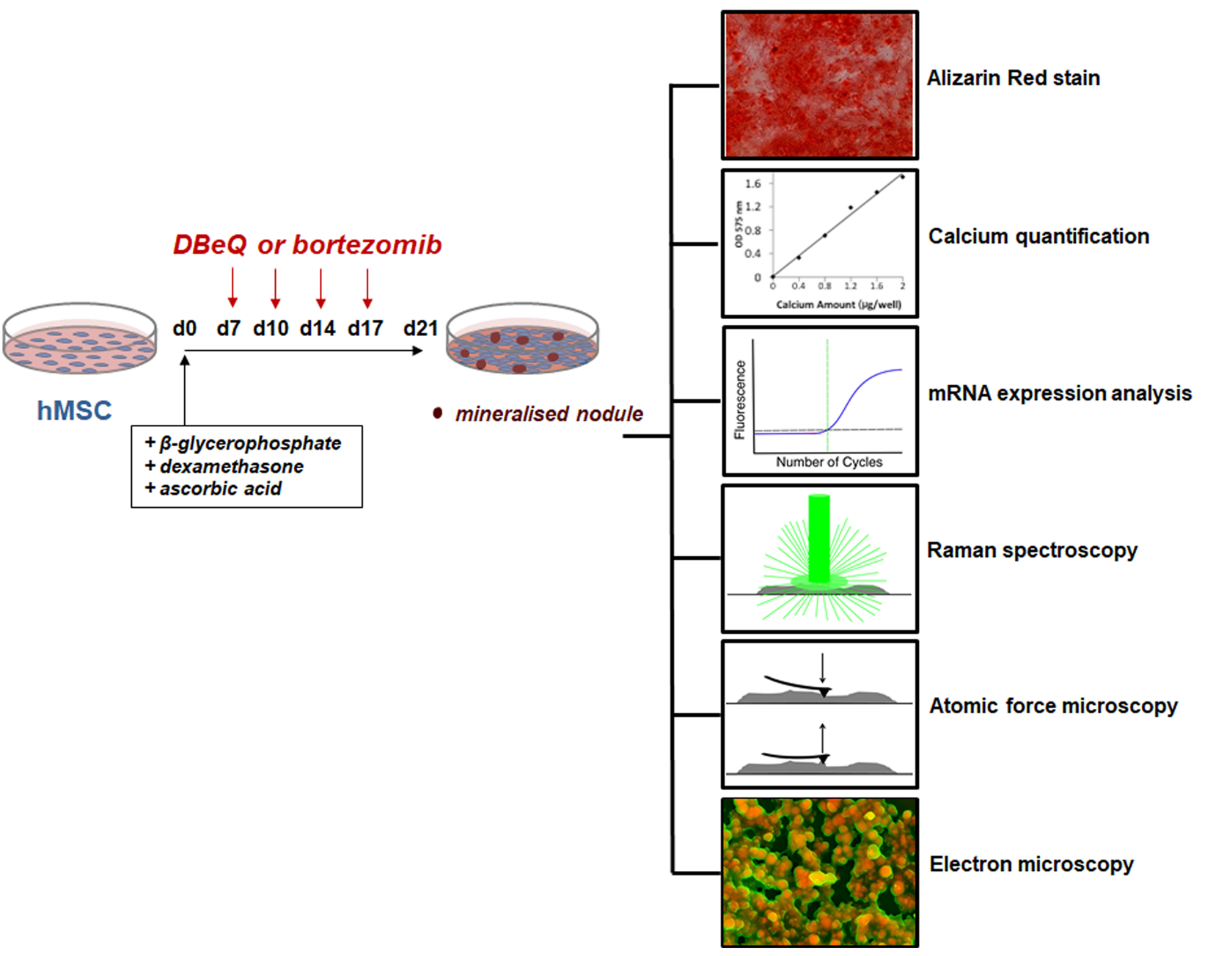
**

**Supplementary Figure 1:** Schematic describing the experimental approach. hMSC were induced to undergo osteogenic differentiation *in vitro*, and DBeQ or bortezomib were added on days 7, 10, 14 and 17 to inhibit VCP/p97 or the proteasome. After 21 days, mineralised cultures were analysed using an array of biological and physical science characterisation techniques.

**
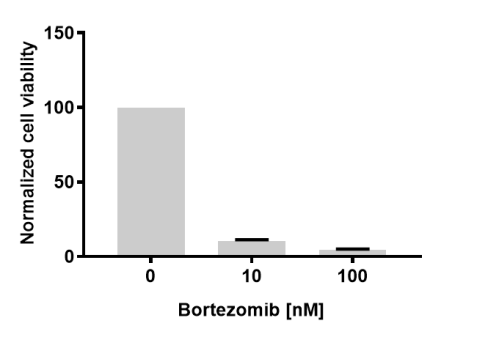
**

**Supplementary Figure 2:** Normalised cell viability of OMP-2 cells treated for 48 h with bortezomib (10 nM and 100 nM) or vehicle control (*n* = 3).

**

**

**Supplementary Figure 3:** Immunoblotting for β-tubulin and ubiquitinated proteins on whole cell extracts from undifferentiated hMSC untreated (control) or treated for 4 h or 24h with DBeQ at 5 μM, bortezomib at 20 nM, or tunicamycin at 5 μg/mL. Another immunoblot from an independent experiment is shown in Figure 1c.

**
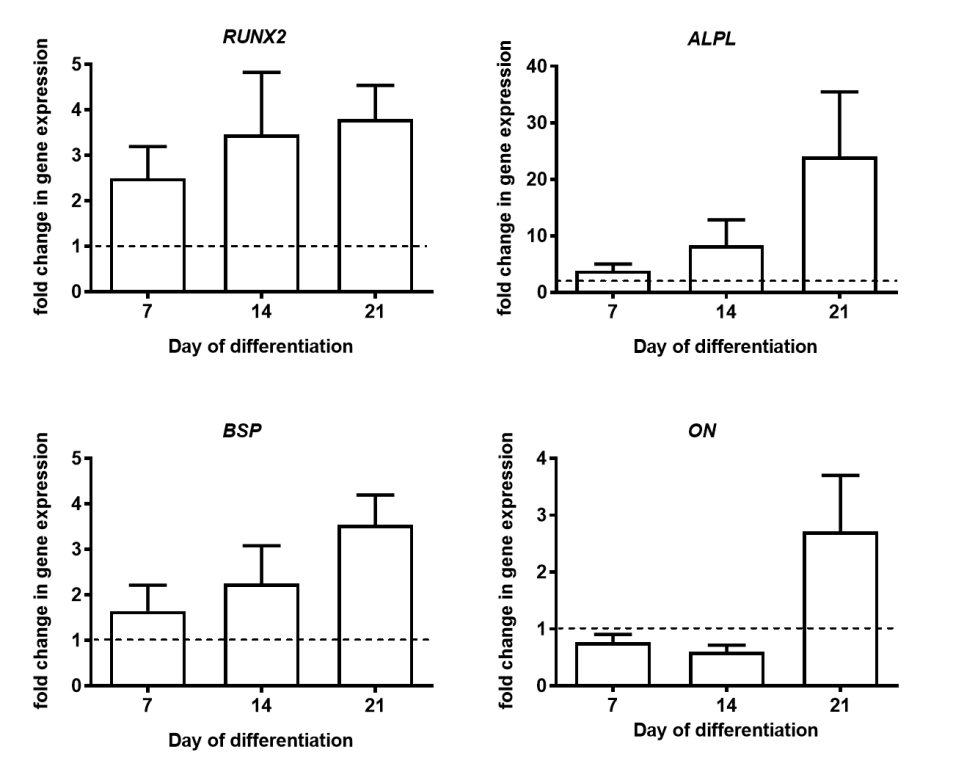
**

**Supplementary Figure 4:** Gene expression analyses (normalised to undifferentiated controls) for markers of osteogenesis (*RUNX2*, *ALPL*, *BSP* and *ON*) in hMSC treated with osteogenic medium for 21 days (control conditions). Plots show mean + SEM (*n* = 3).

**
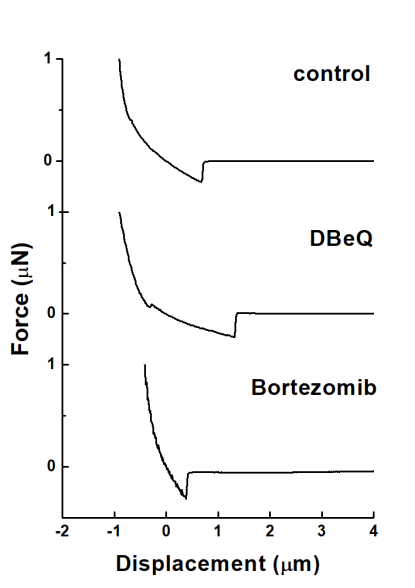
**

**Supplementary Figure 5**: Representative retraction curves generated from AFM-based indentation measurements on mineralised nodules cultured under control conditions or treated with either DBeQ or bortezomib. Adhesion interactions are particularly obvious in the DBeQ-treated group.

**
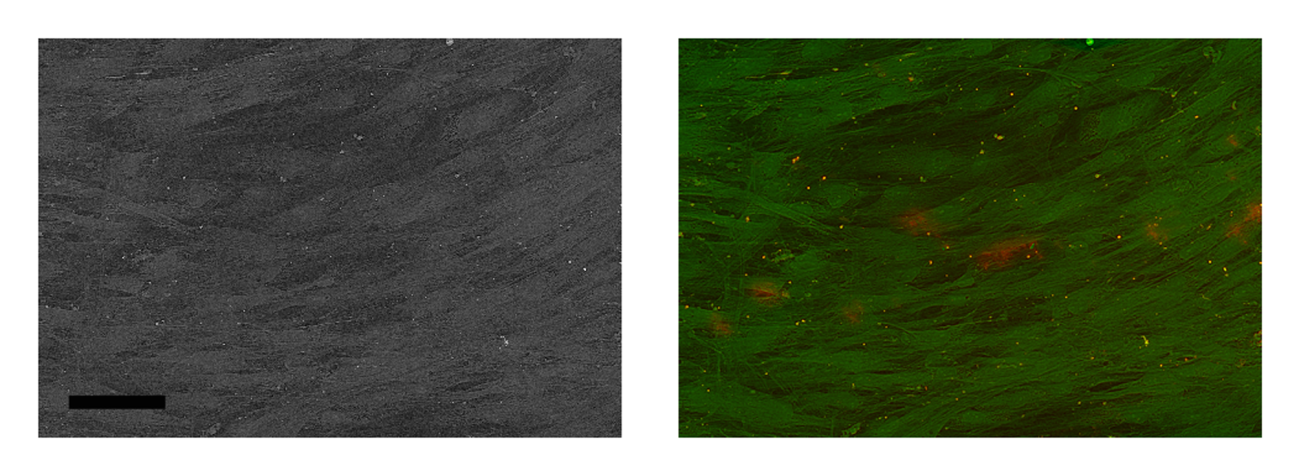
**

**Supplementary Figure 6:** Backscatter and DDC-SEM micrographs of MSC cultured for 21 days under basal conditions. hMSC not treated with osteogenic medium do not form mineralised nodules and DDC-SEM images appear almost entirely green. Scale bar = 100 µm.

**
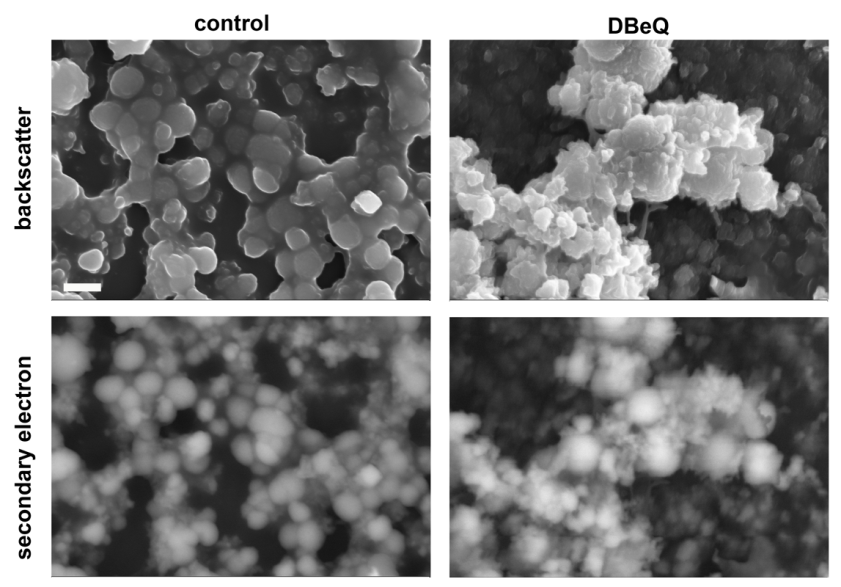
**

**Supplementary Figure 7:** Backscatter and secondary electron images of mineralised nodules collected from control and DBeQ-treated cultures. Images show that whilst control cultures produce mineral that appears smooth, the mineral within DBeQ-treated cultures is often rougher, more plate- or needle-like and indicative of that produced by non-physiological precipitation reactions rather than a controlled biomineralisation process. Scale bar = 1 µm.
